# Urban Green Space and Its Impact on Human Health

**DOI:** 10.3390/ijerph15030445

**Published:** 2018-03-03

**Authors:** Michelle C. Kondo, Jaime M. Fluehr, Thomas McKeon, Charles C. Branas

**Affiliations:** 1 USDA Forest Service, Northern Research Station, Philadelphia, PA 19103, USA; 2 Urban Health Lab, University of Pennsylvania, Philadelphia, PA 19104, USA; tuf55745@temple.edu (J.M.F.); mckeont@pennmedicine.upenn.edu (T.M.); 3 Department of Epidemiology, Mailman School of Public Health, Columbia University, New York, NY 10027, USA; c.branas@columbia.edu

**Keywords:** green space, urban, nature, health, violence

## Abstract

*Background*: Over half of the world’s population now lives in urban areas, and this proportion is expected to increase. While there have been numerous reviews of empirical studies on the link between nature and human health, very few have focused on the urban context, and most have examined almost exclusively cross-sectional research. This review is a first step toward assessing the possibility of causal relationships between nature and health in urban settings. *Methods*: Through systematic review of published literature, we explored the association between urban green space and human health. *Results*: We found consistent negative association between urban green space exposure and mortality, heart rate, and violence, and positive association with attention, mood, and physical activity. Results were mixed, or no association was found, in studies of urban green space exposure and general health, weight status, depression, and stress (via cortisol concentration). The number of studies was too low to generalize about birth outcomes, blood pressure, heart rate variability, cancer, diabetes, or respiratory symptoms. *Conclusions*: More studies using rigorous study design are needed to make generalizations, and meta-analyses, of these and other health outcomes possible. These findings may assist urban managers, organizations, and communities in their efforts to increase new or preserve existing green space.

## 1. Introduction

There have been numerous reviews of empirical studies on the link between exposure to natural green space and human health [1,2,3,4]. These reviews have focused on various topics, including children [5], mental health [6], and violence [7]. However, very few of these reviews have focused on studies that explicitly relate to urban green space, as opposed to nature in any form, despite the fact that over half of the world’s population now lives in urban areas, and this proportion is expected to increase to two-thirds by 2050 [8]. A prior review has provided a general overview of ways in which urban green space is associated with health [9], incorporating almost exclusively cross-sectional research, which has left many unanswered questions regarding the existence of any possible causal relationships between nature and health in urban settings.

Given these gaps in knowledge, this review focuses on studies that have taken experimental, quasi-experimental, or longitudinal approaches. We have excluded cross-sectional studies from this review because they do not permit temporal examination of change in health metrics and outcomes because of changes in green space exposure. Studies of human health responses to urban green space are especially relevant to governments, organizations, and communities that are making efforts to either introduce new or preserve existing green space for residents of urban areas. Urban planning and place-based initiatives are increasingly addressing not only economic and environmental priorities, but also public health goals [10]. Cities are increasingly adopting an urban health agenda [11], which prioritizes the relationship between urban land, natural resources, and human health. These groups need evidence that is relevant to not only land and natural resource management but also health outcomes in urban areas. The purpose of this review is to systematically review studies evaluating the association between urban green space and human health. 

## 2. Materials and Methods 

We conducted a systematic literature review investigating the relationship between urban green space or urban greening interventions, and health. We followed Preferred Reporting Items for Systematic Reviews and Meta-Analyses (PRISMA) guidelines [12] when conducting and reporting this systematic review. The primary question we explored in this review was: What is the association between urban green space and urban health? To answer this question we searched PubMed, Web of Science, PSYCHinfo, and Cochrane databases in January 2018. We restricted searches to the years ranging from January 1976 to December 2017.

We identified articles using the main search terms of urban, green space, and health. We submitted a standard Boolean search phrase, with syntax tailored to each database. In Web of Science our exposure search term was the following: greening OR green space OR greenness OR urban nature OR nature area OR urban garden OR community garden OR open space OR built environment OR park OR public space OR vacant lot OR green infrastructure OR land use OR environment design OR walkability. Our outcome search term was the following: cognitive function* OR attention OR restoration OR rumination OR neural activity OR life functioning OR mood OR stress OR distress OR emotion* OR anxiety OR mental health OR depression OR well-being OR personality OR cardiovascular OR heart rate OR blood pressure OR mortality OR cholesterol OR allerg* OR cortisol OR alpha amylase OR diabetes OR fitness OR physical activity OR MVPA OR exercise OR BMI OR body fat OR general health OR birth outcome* OR smoking OR aggression OR crime* OR safety.

**Figure 1:**
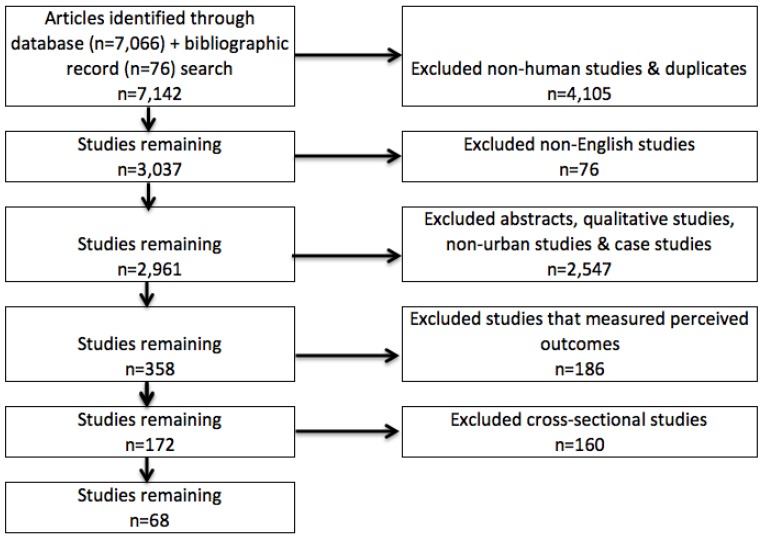
Article Selection Process.

Our search identified studies that measured human health in association with exposure to green space. We only considered studies that were conducted in urban settings, or with participants that were urban residents. We defined urban in contrast to rural, forest, or wildland settings, and included study sites that were within an urban jurisdiction or urban metropolitan area. In addition, we did not include studies based on region- or country-wide samples that did not classify participants as urban residents with separate analyses. Studies of patients admitted to urban hospitals were not included unless patient residential location was coded as urban versus non-urban and was used as a fixed effect (not the reference category) or stratifying variable in statistical models. Studies using perceived measures of the environment (e.g., attractiveness or aesthetics) to estimate exposure, or exposure effect, were excluded. Likewise, studies that tested health response to nature images or pictures were excluded. In cases where urban determination was not clear, the three study authors (Michelle Kondo, Thomas McKeon, and Jaime Fluehr) made a determination by consensus. Figure 1 illustrates our article selection process. 

Our article selection process is illustrated in Figure 1. The initial database searches identified 7067 articles. In addition to electronic databases, we searched bibliographic reference lists of other existing review papers on similar topics and found 186 records that did not result from the database search. The four study authors determined study inclusion criteria and three study authors (MK, TM, JF) made specific inclusion decisions. We first excluded non-human studies and duplicate records, leaving 3037 studies. We then removed non-English studies, which left 2961 studies. Next, we excluded abstracts, qualitative studies, case studies, and non-urban studies leaving 358 studies. We then excluded studies that used subjective perceptions of green space, such as environment “attractiveness” or “aesthetics”, “perceived neighborhood park quality”, “design preferences”, “perceptions of the built environment”, “perceived environment”, “perceived environmental attributes”, “neighborhood perceptions” as the exposure metric unless green space/vegetation was explicitly included in the measure, leaving 172 studies. Each author then reviewed the remaining papers independently to determine eligibility based on these standards, and any disagreement was resolved through discussion. Finally, we excluded cross-sectional studies, or studies that did not compare measures over time, or between treatment and control groups, leaving 68 papers.

We recorded study design, location, years, unit(s) of analysis, sample size, population or sample characteristics, environmental exposure and control exposure, health outcome(s), and direction of findings from each of the studies. We did not conduct meta-analyses of study findings due to inconsistencies in study design, exposure measurements, comparison techniques, and statistical analysis and reporting methods. In the few cases that studies reported estimates that could be grouped, the number of studies was too low to conduct meta-analysis.

## 3. Results

### 3.1. Article Selection

A total of 68 studies met our inclusion criteria. Below, we discuss aspects of study design, location, exposure measurement, health outcomes and study findings.

### 3.2. Study Design and Procedures

Table 1 shows six categories of study design among selected studies. The majority of studies were experimental, using either between- (*n* = 14) or within-subjects (*n* = 21) design. In general, these studies measured response of mental health, cardiovascular systems, metabolic systems, and physical activity to real-time or short-term exposure to natural urban settings versus built urban settings. Many of these studies were able to test for dose-response, by comparing health measurements over time between groups.

Twenty studies were longitudinal, based on either cohort or population samples. These studies examined the association between environmental exposures (usually green space at or near participant residence) and all categories of health outcomes. Nine studies were quasi-experimental, these studies primarily examined association between observed changes among an intervention and control group in cardiovascular, mental health, physical activity and violence outcomes, and nature exposure, after an urban greening intervention. Three studies used a case-crossover study design and one study was a randomized controlled trial (though with small sample size).

### 3.3. Sample Characteristics

The unit of analysis in most cases was the individual, with exceptions including place-based studies of vacant lots [13,14], green stormwater infrastructure sites [15], or tree removals at the Census block group level [16]. In addition, for studies that conducted time-activity monitoring, each location-specific measurement served as the unit of analysis. Table 1 shows that sample size varied widely, ranging from 12 to 3,026,603. Within- and between-subject studies in general had smaller sample sizes. The majority of studies were conducted with both male and female participants. Nineteen of the 69 studies were conducted with youth or adolescent participants [17,18,19,20,21,22,23,24,25,26,27,28,29,30,31,32,33,34,35].

### 3.4. Location

The majority of studies (*n* = 27) were conducted in the United States [13,14,15,16,22,23,24,30,32,34,35,36,37,38,39,40,41,42,43,44,45,46,47,48,49,50,51,52]. The next most frequent study location was the United Kingdom (*n* = 13) [21,25,29,31,33,53,54,55,56,57,58,59,60]. There were five studies conducted each in the Netherlands [20,26,61,62,63] and in Canada [64,65,66,67,68]. Four studies were conducted in Japan [69,70,71,72], and three were each conducted in Australia [73,74,75], and Lithuania [76,77,78]. There were two studies each conducted in Denmark [27,28] and Germany [18,19]. In addition, one study each was conducted in Finland [79], Italy [80], and Spain [17]. Only eight of the studies were conducted in lower-income, “deprived” neighborhoods, with lower-income participants or by stratifying study participants by income [13,14,15,39,40,42,46,60].

### 3.5. Green Space Exposure Measurement

Table 2 shows six categories of green space exposure measurement found among the selected studies.

#### 3.5.1. Green Space Characteristics of Residential Area

The majority of studies used area-based measures to derive individual exposures. In urban green space studies these measures are most often represented as quantity or density of residential green space (*n* = 23). The spatial extent used (for example, to calculate green space density) was typically a political boundary such as ward, statistical area or postal code.

Most studies used a vegetation index derived from satellite imagery, such as the Normalized Difference Vegetation Index (NDVI) to indicate amount of green space surrounding participant residences. NDVI cell values represent percent vegetation, and a median or mean value within a distance-based buffer or administrative boundary can be used (ranging from 30 m to 1 km) [17,18,24,35,39,45,65,66,68,74] though the NDVI value at the residence has also been used [51]. 

Other studies measured exposure as proximity to parks, for example distance from participant residence to the nearest park [19,29,48,78]. Wolch et al. [22] quantified exposure as percent park space within 500 m of residence. Richardson et al. [33] did the same, combined with an indicator of whether or not participants had access to a garden. Other studies combined multiple measurements of green space exposure such as presence, proximity, quality, and area [75], or a subjective scoring of quantity of neighborhood green space [26]. One study indicated exposure as whether or not participants lived near parks, on tree-lined streets, or had a garden on their residential property [72].

While area-based measures are most widely used, they are subject to the uncertain geographic context problem, in which exposure estimates can be affected by the delineation of area units and lack temporal component [81]. There are limitations to assuming exposure is directly related to place of residence because most people do not spend the majority of daytime hours at home, but rather in activity spaces surrounding school or work. 

#### 3.5.2. Green Space Characteristics of Activity Space

Eleven studies measured presence/absence of either green space or park space at location of activity (mostly physical activity) [21,23,25,27,28,29,30,31,32,34,52]. Activity spaces represent the totality of locations that a person visits or undertakes daily activities. This might include residences, school, workplaces, places of recreation, and the transportation routes in-between. Technologies are developing that allow researchers to estimate exposures representative of the actual locations that individuals spend time. For example, study participants can wear global positioning system (GPS) trackers, which record sometimes minute-by-minute geo-coded locations throughout the study period.

#### 3.5.3. Nature Walk/Run

Sixteen studies measured physiological response (either using pre-post comparisons or real-time measurements) to green space exposure via a nature walk, and Butryn and Furst [38] prescribed a nature run. All these studies prescribed a route and/or duration and frequency of walks or run. Location descriptions included urban green space [54,70], urban park [38,52,56,59,69,71,79], urban country park [58], or urban campus [64]. Eight of these studies used a within-subjects design, where health-related outcomes were measured in case participants before, during or after a nature walk, and compared to measurements taken before, during, or after a walk in a control environment, such as an urban shopping street [54], urban [59] or built urban [58,69,70,71] setting, urban industrial [38], blue setting [58], or city center [79]. 

The remaining eight studies used a between-subjects design, where health related outcomes were measured in case subjects exposed to an urban open space preserve [37], urban nature [57], urban park [76], or urban campus [64]. These outcomes were compared to those measured in control participants exposed to a social club or swimming club [56], urban [37], built urban [57,77], urban street [76], or indoor setting [43,64].

#### 3.5.4. Greening Interventions

Twelve studies measured changes in health outcomes measured within proximity to environmental interventions. These interventions included neighborhood-level green space improvements [20,60,61], cleaned-and-greened vacant lots [13,14,40,50], construction of a greenway trail [42], installation of planters [49] or green stormwater infrastructure projects [15], or tree deaths and removals [16]. Treatment or intervention status was most often assigned to participants residing within greening intervention neighborhood(s), while control status was assigned to participants residing in non-intervention control neighborhood(s). South et al. [50] measured real-time physiologic response (heart rate) to exposure (among residents) as being within sight of a cleaned-and-greened vacant lot (versus untreated control lot). Four studies observed health or violence outcomes, using secondary data (e.g., crime occurrence data from police), occurring within proximity to greening interventions or changes [13,14,16,40].

#### 3.5.5. Nature Leisure Experience

Five studies measured physiological response (either using pre-post comparisons or real-time measurements) to green space exposure via a leisure-based nature experience. Beil and Hanes [36] asked case participants to do 20 min of outdoor sitting in forested urban nature preserve, and control participants to do 20 min of sitting in either an urban park, plaza, or shopping mall. Hull and Michael [44] asked participants to spend leisure time in an urban park and control participants to spend leisure time at home. Passmore and Howell [67] asked participants to engage in daytime nature activities and control participants to do anagram puzzles. van den Berg et al. [62] asked case participants to engage in 30 min of outdoor gardening, and control participants to engage in 30 min of outdoor reading.

Carrus et al. [80] is one of few studies that compared health response to green space exposure that varied in quality. They surveyed visitors to four parks or natural protected areas of urban areas with varying levels of biodiversity. Activities or experiences were assigned or prescribed in all studies except Carrus et al. [80], which used a convenience sample from visitors at selected locations.

#### 3.5.6. Residential Relocation

One study [73] measured change in health outcomes associated with perceived access to urban parks before and after residential relocation.

### 3.6. Health Outcomes

Table 3 shows selected studies by major health outcome categories, with specific health outcome measurements.

#### 3.6.1. Birth Outcomes

Birth outcomes could be influenced by maternal green space exposure. Prior research has found that birth outcomes are related to stress [82], social capital [83], levels of physical activity [84], and exposure to pollution [85]. It has also been shown that these pathways are related to green space exposure [86,87]. However, only one study examined association between maternal green space exposure in urban areas, at more than one time point, with birth outcomes. Cusack et al. [39] measured maternal residential green space in metropolitan areas of Texas for 3,026,603 births at 1st, 2nd, and 3rd trimesters and for the total pregnancy. They controlled for known individual and neighborhood confounding factors (e.g., demographic background and smoking status). They did not find consistent effects on birth weight, odds of preterm birth or small for gestational age, however they did find some protective effects of increased residential greenness for mothers with low education, mothers that lived in low-income neighborhoods, or for Hispanic mothers.

#### 3.6.2. Cancer

There is evidence that environmental exposures relate to incidence of cancer. For example, long-term exposure to pollution in the air (from combustion and pesticides, to name a few), has been linked to lung cancer [88]. Green space could mediate this relationship by altering air pollution levels. Only one study that met our study selection criteria estimated association between green space exposure and cancer. Demoury et al. [66] examined whether residential green space exposure related to prostate cancer incidence using a population-based case-control study. Controlling for individual factors (demographic background; family and medical history; smoking, alcohol, diet and physical activity-related behaviors), they found that increased residential greenness was associated with lower risk of cancer.

#### 3.6.3. Cardiovascular

*Background*. Exposure to green space may affect the cardiovascular system by way of mitigating harm (e.g., air and noise pollution and heat), restoring mental capacities (e.g., attention restoration or stress recovery), or building capacities (e.g., physical activity, social connectedness) [3,89,90]. The majority of between- and within-subject design studies measured cardiovascular health outcomes. Gidlow et al. [58], Song et al. [70], Song et al. [69], and Song et al. [71] conducted experimental studies using a within-subjects study design in which all subjects were exposed to both green space and control environments. For all studies, participants were asked to first walk for a set amount of time (between 15 and 25 min) in an urban green space, and then that same amount of time in an urban built space. All of these studies except Gidlow et al. [58] measured heart rate (HR), and all four studies measured heart rate variability (HRV). 

*Design*. Experimental studies using a between-subjects design divided participants in to groups and exposed each group to a different environmental exposure [43,50,57,76]. Brown et al. [57] and Grazuleviciene et al. [76] measured physiological responses to a long-term repeated exposure both using random group assignment (Brown et al. [57]: two 20-min walks per week for 8 weeks; Grazuleviciene et al. [76]: 30 min of walking for 7 days), while Hartig et al. [43] exposed participants to 40 min of walking or sitting. Control environments were built urban areas, indoor seating [43], or residential area with unmitigated vacant lots [50]. Hartig et al. [43] and Grazuleviciene et al. [76] took stationary measurement of HR while participants were seated or resting, before and after exposure. South et al. [50] is the only study to conduct ambulatory measurements of participants’ HR before and after a place-based intervention was conducted (vacant-lot greening). Brown et al. [57] took pre- and post-intervention measurements of BP, HR, HRV as well as autonomic function, risk of cardiovascular disease (CVD), and aerobic fitness. 

*Results*. Most experimental studies measuring HR response found lower HR in urban green space compared to control environments. Song et al. [71], Song et al. [70], and Song et al. [69] found lower HR when subjects walked through an urban park compared to a built urban setting. However, these studies had small sample sizes, and order of study site visit was not randomly assigned. South et al. [50], though also based on a small sample and not controlling for individual characteristics that might influence stress response, found a significant decrease in HR for participants that walked by cleaned-and-greened vacant lots. Grazuleviciene et al. [76], based on a small sample, found that the group that walked in an urban park had greater reductions in DBP and HR, and increases in HR recovery when comparing pre- and post-measurements. Hartig et al. [43] found no statistical difference in BP or HR. Brown et al. [57] found no significant differences in any cardiovascular outcomes. 

Measurement of, and findings on HRV have been mixed. Four studies found that HRV was significantly lower in green environments compared to in control environments using pre-post comparisons [69,70,71]. Significant findings of HF component were in a positive direction [69,70,71], while none that measured LF component found significant (positive) differences. Out of the four studies that measured LF/HF component, three studies found no difference [69,70,71], and one found significant negative difference [69]. Brown et al. [57] and Gidlow et al. [58] found no statistical difference in any HRV indicators.

While studies in general found a negative association between urban nature exposure and HR, these studies were predominantly based on small sample size and did not control for confounding factors. Few consistent measures of HRV have been used, in a small number of studies, and findings are mixed. These findings are bolstered by an increasing number of longitudinal cohort and quasi-experimental studies.

Cardiovascular health outcomes were measured in two longitudinal cohort studies. Paquet et al. [74] found no association, while controlling for individual-level demographic factors, between residential green space (using an aggregated construct) and incidence of hypertension or dyslipidaemia among a cohort of adults living in two metropolitan regions of Adelaide, South Australia. Tamosiunas et al. [78] measured the association between green space exposure (distance from residence to nearest park, and self-reported park use) and markers of CVD among a cohort of residents (ages 45–72) of Kaunas City, Lithuania. This study was able to control for both individual-level demographic and health behaviors (smoking and physical activity). They found an increased risk of fatal and non-fatal CVD for participants (especially men) that lived further from green spaces; and increased risk of non-fatal CVD among non-park users that lived further from green spaces.

Two quasi-experimental studies have examined the association between urban nature-based interventions and change in cardiovascular outcomes. Branas et al. [13] conducted a quasi-experimental study of changes in neighborhood crimes and health outcomes (including rates of hypertension and high cholesterol) that occurred near clean-and-green interventions to vacant lots relative to near untreated vacant lots. They found some significant decreases in high cholesterol across the city near treated lots. In a study of changes in incidence of high blood pressure and high cholesterol near green stormwater infrastructure sites compared to near control sites, Kondo et al. [36] found no changes.

#### 3.6.4. Mental Health

*Cognitive Function*. Aspects of brain function, including cognitive development and attention restoration, may be pathways by which environments affect mental health. Six studies tested the association between urban nature exposure and measures of cognitive function. There was a general finding among five studies in this category [17,46,47,58,79] that exposure to urban nature compared to urban built environments improved multiple measures of cognitive function or development, including attention or attentional capacity and working memory.

Five studies used an experimental approach to measure changes in attention in response to a walk in urban nature versus a walk in a built urban setting [37,47,58,79]. Bratman et al. [37] found negative associations between rumination and subgenual prefrontal cortex activation and exposure environment (90-min walk in urban nature preserve versus built urban walk) and by time (pre- post-comparisons). Participants were urban residents. In a between-subjects study of the association between nature exposure, mood, and attentional capacity [47], participants that walked in urban nature preserve had improved attention and ability to reflect on a life problem, relative to those who walked in an urban built area. 

Gidlow et al. [58] asked participants to take 30-min walks in natural (green), natural with water (blue), and residential control (urban) environments, and took measurements of psychological and physiological stress at baseline, at the end of the walk and 30-min after the walk. They found improved attention and restoration only in the green and blue environments. 

Other studies measured nature exposure via residential location, finding positive effects in general. Dadvand et al. [17] and Kuo [46] measured association between green space characteristics at participants’ residence and attention. Kuo [46] found less attention fatigue among public housing residents assigned to live in housing blocks without nearby nature compared to among those assigned to live in blocks with nearby nature. Dadvand et al. [17] measured association between green space exposure and aspects of cognitive development including working memory and inattentiveness based on repeated measurements over a 1-year period among a cohort of children (ages 7–10) in Barcelona. They found that adding traffic-related air pollution concentrations to statistical models helped explain 20–65% of the estimated positive association between green space (using a composite index) and attention. This is the only study to consider effect modification of air pollution exposure. Hartig et al. [43] found no difference in attention between nature exposure and control groups.

In general, experimental study design has been used to assess the relationship between cognitive function, primarily attention, and green space exposure. While sample sizes have been small and non-random, and studies are subject to biases associated with within- and between-subject designs, a positive association has been found with nature exposure.

*General Health*. Six studies examined the association between self-reported measures of general health and green space exposure. These studies used standardized instruments such as the General Health Questionnaire [55], or the General Health Perception Scale [51]. The General Health Questionnaire was used to measure either “general health” [55] or “mental health” [53]. Half of these studies found positive association between green space exposure and generalized measures of health, while the other half found no association.

Astell-Burt et al. [55] used general health questionnaire to indicate mental health in a British Household Panel Survey with nine annual waves. While adjusting for a wide range of individual-level demographics and health behaviors, they found that association between availability of green space and mental health increased in significance and magnitude for both men and women (depending on level of green space) as they aged. Alcock et al. [53] also used data from the British Household Panel Survey between 1991 and 2008, of participants that moved, introducing a quasi-experimental research design. For each participant Alcock et al. [53] analyzed five annual waves; two years prior and three years after relocation. No control participants were included. Controlling for individual and community-level demographic factors, they found that mental health was significantly improved throughout the post-relocation period among participants that relocated to greener areas.

Brown et al. [57] used a questionnaire to assess participants’ general, mental and physical health. Self-reported mental health improved for the nature walk group compared to the control group that took two walks in a built urban setting. Droomers et al. [61] found no difference in self-reported general health when comparing pre-post measurements among residents of neighborhoods that received green space improvements and residents of neighborhoods that did not receive green space improvements. However, the greening improvement was not standard across intervention neighborhoods, and assessment of improvements was assessed via interviews, which could explain a lack of difference in exposures in intervention and control neighborhoods. Tamosiunas et al. [78] and Wolfe et al. [51] also found no association.

*Mood and Emotion*. Eighteen studies have investigated whether nature exposure relates to mood, using various measures (see Table 2). Most of these studies have found evidence of improved mood or emotion after nature exposure, compared to after exposure to a built urban environment. 

While many of these studies were motivated by the question of whether nature exposure could be used as an effective psychological intervention on clinically-relevant mental health disorders such as depression and anxiety, only one study tested change in mood after therapeutic interventions with a clinical population specifically. Barton et al. [56] recruited persons with a health precondition (mental illness). They found that both self-esteem and mood were significantly improved in the green exercise group (compared to control groups), with a dose-response relationship of time. 

Most other studies measured change in mood after exposures obtained during walks of varying length and frequency, in both urban nature and control environments. Mood improved in nature exposure groups compared to in groups that walked in built urban environments [47,58,69,70,71], walked in indoor settings [64], relaxed in an indoor setting [43], or did anagram puzzles [67]. Brooks et al. [64] added the dimension of season to their walking study, finding no effects. Aspinall et al. [54] was the only study to use mobile electroencephalography (EEG) to monitor emotional experience during experimental exposure process. The study found that participants had more positive emotions and less negative emotions when navigating through urban green spaces compared to built urban spaces. 

Instead of an outdoor walk, van den Berg and Custers [62] subjected participants to a stress task and then exposed them to either 30 min of outdoor gardening or 30 min of indoor reading. Although based on a small, non-random sample, they found that positive mood was improved in the outdoor gardening group only.

Semenza et al. [49] conducted a pre-post study of greening interventions in three neighborhoods. They collected pre- and post- survey measurements of mental health, sense of community and social capital from residents living two blocks from the interventions. Although the study had no control group, they found improvements in all three measures. 

Few studies have tested the relationship between mental health and quality of green space exposure. Tyrväinen et al. [79] examined the psychological (restoration and mood) effects of visits to urban environments (urban park, urban woodland or built urban). Restoration and mood improved in both nature settings, but restoration was more improved in the urban woodland. Carrus et al. [80] found an association between biodiversity of green space and well-being, mediated by length of park visit and perceived restorativeness.

Three studies found no difference in association between nature- and control-group exposures and mental health. Butryn and Furst [38] measured mood and feeling states before and after participants, female distance runners, went on a 4-mile run on a natural and a built urban route. In general, they found that though participants preferred the urban park run, mood and feeling states improved post-run in both settings. Hull and Michael [44] asked participants to spend leisure time in an urban park and then in their home, and found improved mood after leisure time in both locations. Grazuleviciene et al. [76] found that negative affect did not change for the nature walk group.

Compared to studies of cognitive function outcomes, a wider variety of study designs have been used to assess the relationship between urban green space exposure and mood. Although mood has been measured using an inconsistent set of scales and concepts, in general a positive association has been found.

*Depression*. Six studies examined the association between green space exposure and depression or depressive symptoms and produced mixed results. Gubbels et al. [20] and Semenza et al. [49] were both greening intervention studies, and both used the Center for Epidemiologic Studies Depression Scale (CES-D). Gubbels et al. [20] conducted a two-wave longitudinal study of changes in walking and cycling frequency associated with neighborhood green space interventions and perceived greenery. This is one of few studies to focus on “deprived” neighborhoods. They found that perceptions of greenery improvements were associated with a decrease in depressive symptoms among adults. Semenza et al. [49], though under-powered, found no difference in depression scale between intervention and control sites. 

The remaining studies tested mental health response to short-term nature exposure (compared to built urban or indoor exposure) through nature walk or run. Butryn and Furst [38], Song et al. [70], and Song et al. [69] used the Profile of Mood States (POMS) questionnaire, and found a negative association between urban green space exposure and depression. While Brooks et al. [64] used the short-form version of the Depression and Anxiety Stress Scales (DASS-21) and found significant time and season effects.

*Stress*. Stress response to urban nature exposure has been measured using psychological and physiological measures. Three studies measured the association between urban green space exposure and psychological stress. All these studies indicate a positive association. Using a within-subjects study design, Beil and Hanes [36] found significant improvements in post-exposure measurements of self-reported stress for participants (especially women) exposed to natural urban settings compared to built settings. Branas et al. [13] also found improvements in self-reported stress among residents living near cleaned-and-greened vacant lots compared to residents living near control untreated vacant lots. Kondo et al. [36] found no difference in self-reported stress among residents living near green stormwater infrastructure projects compared to residents living near wait-list control sites.

There is mixed evidence of an association between urban nature exposure and physiological stress response. Cortisol concentration, measured either by saliva or blood, is a common marker of physiological stress, and was used in five experimental studies [36,58,62,76,79]. It should be noted that none of the studies measured diurnal cortisol concentration. In general, cortisol saliva samples were taken before and after outdoor exposures, though some studies also collected samples during the exposure [62]. Most of these studies found no significant difference between pre- and post- exposure measurements of cortisol [36,58,76,79]. Tyrväinen et al. [79] is one study that examined both the physiological (cortisol concentration) and psychological (restoration and mood) effects of visits to urban environments (urban park, urban woodland or built urban). Cortisol concentration decreased in all three settings. One study [62] found that cortisol concentrations were improved with urban green space exposure (in the outdoor gardening intervention group and not in the indoor reading control group).

*Behavioral Problems*. Only one study examined the association between nature exposure and social, emotional and behavioral problems. Richardson et al. [33] conducted a longitudinal cohort study of children between 2005 and 2010. Participants were approximately 1 year old at recruitment. Using the Strength and Difficulties Questionnaire, and controlling for many individual, family and neighborhood factors, the study found that lack of private access to gardens was associated with hyperactivity-, peer-, conduct-problems and total difficulties, while increasing exposure to green space (measured as percent green space and parks within ward of residence) was associated with improved social outcomes. 

#### 3.6.5. Metabolic

Urban green space exposure could impact weight status or other measurements of the metabolic system via cardiovascular, mental health and physical activity pathways. Seven studies measured body-mass index (BMI) [22,24,51,57,78,80] or other measurements of weight status, including abdominal obesity [74], in association with urban green space exposure. 

Only two of these studies found a negative relationship between BMI and urban nature exposure. Bell et al. [24] conducted a longitudinal cohort study of children ages 3–16 years recruited from a primary care clinic of a health network in Indianapolis, IN. Two BMI measurements were taken at least two years apart and compared with the amount of residential green space (NDVI within 1 km of residence). While controlling for residential density in addition to demographic factors, they found that higher residential green space was associated with lower BMI (z-scores) in the second measurement. Wolch et al. [22] examined the association between proximity to parks with childhood obesity using data from eight annual survey waves from a longitudinal cohort study of 3173 children in California. While controlling for multiple potential confounding factors, BMI growth at age 18 was inversely associated with park access (park acres within 500 m of residence), more so for boys than for girls. 

The remaining five studies found no association between BMI and green space exposure. Brown et al. [57] found no difference between short-term (8-week) green space exposure and BMI and predicted aerobic fitness. The remaining studies used a longitudinal cohort design. Gose et al. [19] examined the association between neighborhood social and environmental factors and BMI using a 4-year longitudinal cohort study of children in Kiel, Germany. They found no difference between long-term green space exposure (distance to nearest park/green space within 800 m of residence) and BMI. Michael et al. [80] found no relationship between neighborhood environment (proximity of park or green space) and BMI among older women over the 18-year study period. 

Paquet et al. [74] found no association between availability/access to public open space (including greenness) and abdominal obesity. However, they found that larger public open space (not necessarily vegetated/green space) was associated with lower risk of developing prediabetes/diabetes. Tamosiunas et al. [78] found that distance from residence to park space was not associated with BMI, however they found that self-reported park users, compared to non-park users were less likely to be obese (BMI greater than or equal to 30).

The majority of evidence suggests no association between BMI and urban green space exposure. However, none of the studies reviewed explored physical activity, cardiovascular disease or mental health, which are expected to be pathways of association, as effect modifiers. 

#### 3.6.6. Mortality

Studies of the relationship between green space exposure and mortality pose strong evidence of consistent negative association. Six longitudinal studies have been conducted with strictly urban cohorts on the association between mortality and green space exposure [51,65,68,72,78]. Although each of these studies was conducted with sub-populations and therefore results should not be generalized, all found that increased green space exposure was associated with reduced odds of mortality. Takano et al.’s [72] foundational study, a prospective cohort of elderly Tokyo residents, found that having green space and gardens nearby one’s residence increased survival odds. Wilker et al. [51] conducted a longitudinal cohort study of adults admitted for acute ischemic stroke between 1999–2008. Using death records collected in 2012, they found a lower hazard ratio for stroke mortality among patients with higher green space exposure (NDVI at residence). 

Two of these longitudinal studies controlled for ambient air pollution concentrations. Villeneuve et al. [68] conducted a cohort study of adults living in 10 urban areas of Ontario, Canada between 1982–86. Among participants, higher green space exposure (NDVI within 500 m of residence), while controlling for ambient air pollution, was associated with reduced non-accidental mortality, with strongest association for respiratory disease mortality. However, this study did not control for smoking behavior. Crouse et al. [65] conducted a large cohort study of mortality among non-immigrant Canadians residing in 30 cities between 2001 and 2011. Using annual measurements of residential green space, they found a protective association with non-accidental, cardiovascular (plus diabetes), cardiovascular, ischemic heart disease, cerebrovascular, and respiratory mortality. Sex, age, income, educational attainment and marriage status modified the estimates.

While not strictly an urban sample, James et al. [45] used mortality data from a cohort of nurses in 11 states of the USA, 84% of whom were living in urban areas. They found that living within the highest quintile of residential greenness reduced risk of overall mortality by 12%. They found reduced risk specifically for cancer, respiratory and kidney disease mortality, and no association with other causes including coronary heart disease. Stratifying by urban versus non-urban residence did not change estimates, and physical activity, air pollution, and diagnoses of depression modified the estimates.

In a single case-crossover study, Gronlund et al. [41] examined how individual and neighborhood characteristics (including percent green space) modified the association between extreme heat (EH) events and cardiovascular mortality using death records from eight cities in Michigan between 1990 and 2007. The odds of cardiovascular mortality were higher among individuals living in zip codes with low amounts of green space.

A growing number of longitudinal cohort studies provide evidence of a significant negative association between green space exposure and mortality. However, while there was more than one study examining (and finding negative association for) all-cause (non-accidental), cardiovascular and respiratory mortality, there was only one study examining other mortality causes, including stroke, cancer, cerebrovascular disease, and kidney disease. It should be noted that most of these studies were conducted with sub-populations and did not control for other environmental exposures (e.g., air pollution) that might affect mortality risk.

#### 3.6.7. Physical Activity

Whether or not someone engages in physical activity may be influenced not only by individual characteristics, but also by the accessibility, features, condition, and actual and perceived safety of their surrounding physical environment [91]. Increased exposure to urban green space could improve health by increasing opportunities and actual physical activity levels. A variety of methods have been used to test the association between green space exposure and physical activity, including momentary/time-activity studies, experimental design (within- or between-subjects), longitudinal studies, and place-based interventions. 

Physical activity is most often measured using accelerometer as time spent in moderate-to-vigorous physical activity (MVPA). Twelve studies examined the association between green space exposure and physical activity, measured as MVPA. Nine of these studies focused specifically on youth or adolescents [21,23,25,27,28,29,31,32,52]. All tracked location of MVPA using a combination of wearable GPS and accelerometer, except de Vries et al. [26] that used activity diaries. Characteristics of momentary locations were typically classified using land use or landcover categories. 

In general, these studies found that amount of time spent in MVPA versus not was higher when surrounded by green space (of differing types). Half of these 12 studies calculated likelihood of MVPA using means comparison, and therefore did not control for confounding factors. Coombes et al. [25] found that the mean number of minutes of MVPA was higher in green space locations than in buildings and paved areas [25]. Jones et al. [21] found that among urban participants, MVPA was more common in gardens and streets than in other environments. Moore et al. [31] found some MVPA in green space, but less than in other domains (school, home, street). Lachowycz et al. [29] found that up to 30% of MVPA among 10–11-year-olds was done in parks. Klinker et al. [27] and Klinker et al. [28] found no significant difference in MVPA for participants when spending time in green space.

The remaining six of these studies used statistical models to calculate odds of MVPA while controlling for individual and community factors. Nearly all these studies found that time spent in urban green space was positively associated with MVPA. Odds of MVPA were higher for study participants when spending time in parks than in other land uses [32,34,59]. de Vries et al. [26] found that adjusted odds of MVPA were higher with higher quantity of residential green space (using subjective scoring). Almanza et al. [23] found that MPVA was nearly five times greater among children that spent more than 20 min of time in green space, than among children with no green space exposure. 

Finally, Zenk et al. [52] found no association between presence of park land use and MVPA. It should be noted that none of these time-activity monitoring studies, except Zenk et al. [52], coded GPS data by activity spaces or transportation modes, which could limit causal inference [92]. Likewise, instead of MVPA, Grazuleviciene et al. [77] compared exercise duration between participants that walked in an urban park and participants that walked in a built urban environment, and found no difference.

Longitudinal studies have found positive association between green space exposure and self-report measures of physical activity. Beenackers et al. [73] found that after residential relocation, participants’ self-reported cycling behavior increased, moderated by self-reported access to parks. Sugiyama et al. [75] conducted two surveys of physical activity four years apart of a cohort in Adelaide, Australia. They found that walking maintenance was associated with green space exposure (total and largest area within 1.6 km of residential neighborhood). Tamosiunas et al. [78] found that inactive participants were more likely to be non-park users and to have less exposure to green space.

Five studies evaluated physical activity in response to a greening intervention, and there is mixed evidence of impact. Branas et al. [13] found a positive association between exercise levels (increase) in residents living near vacant-lot cleaning-and-greening interventions. Thompson et al. [60] included a small sample, however they found an increase in exercise frequency among surveyed residents of a greening intervention neighborhood compared to control area. In addition, Gubbels et al. [20] conducted a two-wave longitudinal study of changes in walking and cycling frequency associated with neighborhood green space interventions and perceived greenery. This was one of few studies to focus on “deprived” neighborhoods. They found that residents of greening intervention neighborhoods had a smaller decrease in cycling among adolescents compared to residents of control neighborhoods. 

The remaining studies found no impact. Using five years of cross-sectional surveys Droomers et al. [61] found no change in physical activity among residents of intervention neighborhoods compared to among residents of control neighborhoods. Although conducted with a small sample, Gustat et al. [42] found no difference in rate of physical activity (including MVPA) among residents of a single greening intervention neighborhood and two control neighborhoods. 

A wide variety of methods have been used to test the association between green space exposure and physical activity. While in general, experimental (within- or between-subjects) and longitudinal studies have found a positive association between urban green space exposure and physical activity, findings from place-based intervention (observational) studies have found no association. 

#### 3.6.8. Respiratory

Only one study has been conducted on the association between respiratory symptoms and urban green space exposure, with beneficial effects found for more green space exposure. Fuertes et al. [18] compared respiratory outcomes associated with green space exposure (average NDVI within 500 m of residence) among two 10-year longitudinal cohorts with identical study designs; one urban and one rural cohort. They found an increased association between allergic rhinitis and eye and nose symptoms only among the urban cohort, and risk estimates increased with increasing air pollution concentrations.

#### 3.6.9. Violence

Studies that have examined the association between urban green space and violence were mostly quasi-experimental, with analysis of change in violence after a greening intervention at both intervention and control locations. Violence-related outcomes included crime, feelings of safety and aggressive behaviors. Three studies that measured changes in crime found significant decreases after place-based green space interventions occurred [13,14,36]. In a study of changes in crime outcomes near 4,436 cleaned-and-greened intervention vacant lots compared to near 13,308 control lots, Branas et al. [13] found reduced gun violence, though no difference in nuisance or drug-alcohol crimes, near greened vacant land compared near blighted vacant land. Kondo et al. [14] examined the association between changes in crime around 254 intervention cleaned-and-greened (some by community members and some by city-contractors) vacant lots in Youngstown, Ohio compared to around 959 control lots. The study found a reduction in all crimes around intervention lots, specifically in property crimes around contractor-greened lots and in violent crimes around community reuse lots. In addition, Kondo et al. [36]’s quasi-experimental study of the association between green stormwater infrastructure installation and crime did not find any significant effects on violent crimes but did find reduced narcotics possession arrests around greened sites compared to control locations. 

A single study used the removal of trees as a natural experiment of the relationship between green space and crime. Kondo et al. [16] used quasi-experimental design to examine the change in crime in Cincinnati after the emerald ash borer decimated the ash tree population and found an association between removals and Census-block levels of both violent and property crimes. In addition, there has been one experimental study, a small-sample randomized controlled trial of violence outcomes associated with a vacant lot greening intervention [40]. After the intervention, residents living near intervention lots felt safer compared to residents living near lots that were left vacant. All these studies featured greening interventions (and controls) occurring primarily in low-income neighborhoods.

Using both a case-control and case-crossover technique, Kondo et al. [30] examined the presence versus absence of tree cover for 1-min activity pathpoints on the day of assault, and its influence in risk of assault. They found that being under tree cover reduced odds of gun assault using case-control and case-crossover methods, especially in low-income neighborhoods.

In addition, Younan et al. [35] examined the association between violent behavior (aggression) and urban green space exposure (average NDVI surrounding residence) using a longitudinal cohort study with four waves. They found that increased exposure to green space was associated with reduced aggressive behaviors.

Seven studies have examined the association between urban green space and violence outcomes, and most have been quasi-experimental studies comparing pre- and post-intervention levels of crime or violence between treatment and control groups. Although nearly all are lacking true randomization, all, but one, found significant negative relationships.

## 4. Discussion

Over half of the world’s population now lives in urban areas, and this proportion is expected to increase. While there have been numerous reviews of empirical studies on the link between nature and human health, very few have focused on the urban context, and most have examined almost exclusively cross-sectional research. This review is a first step toward assessing the possibility of causal relationships between nature and health in urban settings. 

An increasingly varied range of study designs is being employed to assess the urban nature-human health relationship. Many are observational, including case-control or case-crossover methods, and prospective or other longitudinal cohorts most recently have been taking advantage of large-sample health surveys. Quasi-experimental studies have examined the association between changes in the urban environment, usually an increase in green space, on health outcomes. 

Experimental studies have largely tested the effects of urban nature exposure (in the form of walks or activity) in contrast with exposure to built urban settings on short-term health outcomes. However, it should be noted that randomization is often not incorporated in these studies. As of December 2017, there have been no experimental studies published, including randomization, of the effects of greening interventions on human health or safety. Time-activity monitoring allows micro-scale exposure assessment, which could minimize exposure misspecification. Yet it is important to account for daily activity spaces and transportation modes [92].

The range of health outcomes included in these urban studies is not as varied as found in the broader literature on nature-health. For example, we found only one study each of green space effects on birth outcomes, cancer and respiratory symptoms, and we found very few on stress. Findings in all health outcome categories were mixed. However, we found consistent negative association between urban green space exposure and mortality (all cause, cardiovascular and respiratory), measurements of heart rate (short-term), and violence, and positive association between urban green space exposure and attention and mood. Though many studies relied on simple means comparison, and therefore were not adjusted for confounding factors, there was consistent positive association found between green space exposure and physical activity (measured as MVPA). These patterns could be affected by publication bias, in which positive (or in this case, negative) findings are more likely to be published in peer-reviewed literature [93].

In most cases, it is not possible to observe patterns of findings of association between urban green space exposure and health outcomes. The number of studies is too low to make generalizations about birth outcomes, blood pressure, heart rate variability, cancer, diabetes, or respiratory symptoms. Results were mixed, or no association was found in general, in studies of urban green space exposure and general health, depression, and stress (via cortisol concentration). One potential reason for such mixed results is lack of consistency in the definition of urban nature, and measurement of its exposure, is necessary. The terms green space and nature are often used without precision, and in some cases refer to nature in general, but sometimes refer to urban vegetation [94]. Exposure to urban nature has largely been measured via characteristics of residential environments or activity spaces using various area-based measurements (e.g., density of green space within an administrative boundary), and is subject to the uncertain geographic context problem [81]. However, recent advancements in exposure measurement suggest that exposure to green space is more complicated than, and perhaps misrepresented by, land use and landcover datasets. More accurate and dynamic green space exposure measurement among urban populations will take in to account aspects other than presence/absence of tree canopy, but also include aspects of access (in all of its physical, cultural and political forms), quality, quantity, and temporality [81,86,95]. More consistency in exposure measurement will be required to build quantitative meta-analyses.

Studies of the association between urban green space exposure and health could be improved by emerging technologies that quantify exposure to green space. Methods to assess environmental, or external, exposures have advanced along with methods to assess the exposome, in general, as a tool to detect causes of disease [96]. Quantitative measurements combined with qualitative descriptions, shown in multiple dimensions, of experimental exposures could prove to be useful [94]. An increasing number of technologies that produce geocoded quantitative descriptions of the environment will provide tools to understanding the relationship between urban green space and health. Increasing resolution and detail of remote sensing images will provide more accurate place-based information about presence/absence and perhaps species of vegetation, however it may not provide data about access or quality of green space. Comprehensive sets of images of locales are increasing, such as those provided for mapping and way-finding such as Google Earth, and could be used for historic characterizations and analyses of urban environments. In addition, portable and personal sensing technology, sometimes aided by smart phones, is developing that may, with increasing accuracy, measure both geo-located environmental exposure and health data [97]. These sources, combined with administrative and crowd-sourced datasets, could increase ability to represent access to green space as it plays out within urban residents’ everyday patterns and experiences, beginning with the type, size, quality and distance of urban green spaces [98].

It should also be noted that the experimental studies, for example using a between- or within-subjects design, due to reasonable constraints, were not conducted with random sampling of the population. Rather, most were conducted with university students, sometimes of one sex. While the recruitment inclusion/exclusion criteria can rule out confounding factors such as medical pre-conditions, pre-post comparisons of health metrics in most of these studies did not control for potential confounders. Individual differences can be a confounder in between-subjects studies; while within-subject studies can be biased by environmental differences (e.g., climate at each study site) and order effects. Care should be taken in generalizing results.

Generalizability is also limited because neither urban areas, nor urban populations are homogenous. The majority of the studies included in this review were conducted in the global North. There is a need for research on the nature-health connection in cities of the global South. Most studies were done with adult participants, while relationships could differ by age, sex, socioeconomic status, racial or ethnic background, or other factors such as disease precondition. In addition, very few studies focus on health inequalities, or the impact of urban nature specifically on disadvantaged or vulnerable populations. Moving forward, it will be important to conduct studies, with care, specifically with vulnerable populations, in high-risk communities, to assess the role that green amenities play in a complex set of structural inequalities. Such research could uncover necessary and cost-effective solutions to public health issues that pose immense cost to individuals, communities, and nations [95,99].

Finally, urban planners and public health professionals need evidence of the impacts of specific therapeutic or place-based interventions to help address public health issues facing their constituents. There is a need for more (randomized, controlled) intervention studies to assess which specific changes to urban environments, or to the everyday habits and routines of urban residents, are needed to make a difference to chronic disease-, injury- and violence-related outcomes.

## 5. Conclusions

This review of experimental, quasi-experimental, and longitudinal studies found evidence of a positive association between urban green space and attention, mood, and physical activity, and negative association with mortality, short-term cardiovascular markers (heart rate), and violence. More studies using rigorous study design are needed to enable generalizations, and meta-analyses, of these and other health outcomes. These findings may assist urban managers, organizations, and communities in their efforts to increase new or preserve existing green space.

## Figures and Tables

**Table 1:** Description of study sample populations, stratified by study design.

**Citations**	**Sample Size**	**Mean Age**	**Sex**	**Population**
**Between-subjects design (*n* = 14)**				
Barton et al., 2011	53	53	both	Adults with mental health problems
Bratman et al., 2015	38	27	both	Adults residing in urban areas
Brooks et al., 2017	121	22	both	University students
Brown et al., 2014	100	42	both	Adults; employees of a UK-based company
Carrus et al., 2015	569	41	NDR	Adult visitors to study sites
Grazuleviciene et al., 2015	20	63	both	Adults (ages 45–75); residents of Kaunas city with cardiopulmonary disease
Grazuleviciene et al., 2016	20	63	both	Adults (ages 45–75); residents of Kaunas city with cardiopulmonary disease
Gustat et al., 2012	473	44	both	Adults; residents of intervention and comparison neighborhoods
Hartig et al., 1991	34	20	both	University students
Jones et al., 2009	100	DNR	both	Youth (ages 9–10)
Kuo 2001	145	DNR	DNR	Adults; residents of Robert Taylor Homes, Chicago, USA
Mayer et al., 2009	76	DNR	both	University students
South et al., 2015	12	DNR	both	Adults living within 2 blocks of study lots
van den Berg and Custers 2011	30	58	both	Adult members of allotment complex
**Case-control or Case cross-over (*n* = 3)**				
Demoury et al., 2017	3927	64/65	male	Prostate cancer patients and population controls
Gronlund et al., 2015	DNR	DNR	DNR	Decedents of all-natural, heat-related, cardiovascular, and respiratory cause mortality
Kondo et al., 2017	409	18/19	male	Boys and men (ages 10–24 years) who had been gun assaulted and community controls
**Quasi-experimental (*n* = 9)**				
Alcock et al., 2014	1064	38	both	Individuals who relocated to a different residential area
Beenackers et al., 2012	1427	40	both	Adults moving in to new housing developments
Branas et al., 2011	4436	NA	NA	Vacant lots
Droomers et al., 2016	48,132	DNR	both	Respondents to Dutch National Health Interview Survey
Kondo et al., 2015	238	NA	NA	GSI eligible and project sites
Kondo et al., 2016	244	NA	NA	Vacant lots
Kondo et al., 2017	307	NA	NA	Block groups infected with EAB
Semenza et al., 2006	265	DNR	both	Residents living within 2 blocks of study sites
Thompson et al., 2013	215	DNR	both	Adults (ages 16+)
**RCT (*n* = 1)**				
Garvin et al., 2012	21	46	both	Adults
**Citations**	**Sample Size**	**Mean Age**	**Sex**	**Population**
**Longitudinal cohort (*n* = 20)**				
Astell-Burt et al., 2014	65,407	DNR	both	Adults (ages 15+) surveyed in British Household Panel Survey
Bell et al., 2008	3831	DNR	both	Youth (ages 3–16) residents of Marion County
Crouse et al., 2017	1,170,343	DNR	both	Adult (ages 19+); non-immigrant, urban Canadians deceased or not deceased
Cusack et al., 2017	3,026,603	NA	both	Mothers living in metro areas of Texas
Dadvand et al., 2015	2593	9	both	Youth (ages 7–10); school children in Barcelona
Fuertes et al., 2014	5803	DNR	both	Youth (ages ≤ 10); birth cohorts from Munich or Ruhr area, Germany
Gose et al., 2013	485	6	both	Youth (ages 9–11); residents of Kiel, Germany
Gubbels et al., 2016	401/454	13/46	both	Adolescents/Adults; residents of deprived urban districts in Netherlands
James et al., 2017	108,603	69	female	Registered nurses (ages 30–55 in 1976) from 11 USA states
Michael et al., 2014	2003	146	female	White, non-Hispanic women (ages 65+) living in four metropolitan areas of USA
Paquet et al., 2014	3205	DNR	both	Adults (ages 18+); residents of Adelaide, Australia
Richardson et al., 2017	2909	5	both	Youth (age 12 mo. in 2005) residents of urban Scotland
Sugiyama et al., 2013	1036	49	both	Adults; residents of Adelaide, Australia
Tamosiunas et al., 2014	5112	60	both	Adults (ages 45–72); residents of Kaunas city
Takano et al., 2002	3144	DNR	both	Adults (born in 1903, 1908, 1913, or 1918); residents of Tokyo metro area, Japan
Villeneuve et al., 2012	575,000	DNR	both	Adults (ages 35+); residents of urban Ontario, Canada
Wilker et al., 2014	1645	73	both	Patients admitted for acute ischemic stroke
Wolch et al., 2011	3173	DNR	both	Youth (ages 9–10); residents of southern CA, USA
Wolfe et al., 2014	1112	64	both	Individuals with medically diagnosed somatic chronic disease
Younan et al., 2016	1287	DNR	both	Youth (ages 9–18); monozygotic and dizygotic twin pairs who had at least two assessments of aggressive behaviors
**Within-subjects design (*n* = 21)**				
Almanza et al., 2012	208	DNR	both	Parent-child (ages 8–14) pairs; residents of Chino, CA, USA
Aspinall et al., 2015	12	31	both	University students
Beil and Hanes 2013	15	42	both	Adults; residents of Portland metro area, OR, USA
Butryn and Furst 2003	30	31	female	Non-elite distance runners
Coombes et al., 2013	100	DNR	both	Youth (ages 9–10); school children in Norfolk, England
de Vries et al., 2007	422	8	both	Youth (ages 6–11); residents of deprived urban neighborhoods in Netherlands
Gidlow et al., 2016	38	41	both	Adults (ages 18+); living, working or studying in West Midlands, UK
Hull and Michael 1995	108	22	both	Park visitors
Klinker et al., 2014a	192	13	both	Youth (ages 11–16); school children in Haraldsgade district, Denmark
Klinker et al., 2014b	170	13	both	Youth (ages 11–16); school children in Haraldsgade district, Denmark
Lachowycz et al., 2012	902	12	both	Youth (ages 10–11); school children in Bristol, England
**Citations**	**Sample Size**	**Mean Age**	**Sex**	**Population**
Moore et al., 2014	28	12	both	Youth (ages 11–14); school children in Middlesbrough, England
Oreskovic et al., 2015	80	13	both	Youth (ages 11–14); non-Hispanic white and black, and Hispanic who sought care at a community health or recreation center and resident of Boston area, MA, USA
Passmore and Howell 2014	84	21	both	University students
Rodríguez et al., 2012	145	16	female	Youth; school children in 8th grade in San Diego and Minneapolis/St. Paul metro areas, USA
Sellers et al., 2012	40	23	both	University students
Song et al., 2014	17	21	male	University students
Song et al., 2015	23	22	male	University students
Song et al., 2013	13	23	male	University students
Tyrväinen et al., 2014	77	DNR	both	Adults; working in Helsinki, Finland
Zenk et al., 2011	120	DNR	both	Residents of three areas of Detroit, MI USA

Table 1 Definitions: DNR: did not report; NA: not applicable; Between-subjects: Experimental design in which two or more groups are tested by a different factor (or environment) simultaneously; Case cross-over: Etiologic design in which cases serve as their own controls, and effects of case vs. control exposures on health outcomes can be compared. Longitudinal cohort: Observational design in which data is gathered for the same subjects over time. Quasi-experimental: Similar to experimental design but lacking random assignment to treatment or control group. Randomized-controlled trial (RCT): Experimental design in which treatment and control groups, randomly assigned, are compared before and after an intervention or exposure occurs. Within-subjects: Experimental design in which two or more groups are exposed to each study condition.

**Table 2:** Green Space Exposure Measurement.

**Green Space Exposure Type**	**Exposure Details**		
	**Case/Intervention Group or Cohort**	**Control Group(s)**	**Studies**
**Green space characteristics of activity location (*n* = 11)**			
	Urban green space at location of activity (pathpoint)		[21,23,27,28,29,30,31]
	Park land use at location of activity (pathpoint)		[25,32,34]
	Percent park land use within residential neighborhood and activity space		[52]
	Parks and tree-lined streets near the residence; garden at the residence		[72]
**Green space characteristics of residential area (*n* = 23)**			
	Average NDVI within proximity of residence		[18,22,24,35,39,45,51,65,66,68,74]
	Percent green space within administrative boundary of residence		[41,53,55,63]
	Distance from residence to nearest park or green space		[19,29,48]
	Percent natural space and parks within 500 m of residence; garden access		[33]
	Surrounding greenness index: Average NDVI within 250 m of residence, 50 m of home-school commute, and 50 m of school		[17]
	Subjective scoring of quantity of neighborhood green space and water		[26]
	Residence in public housing units with vegetation	Residence in public housing units without vegetation	[46]
	Presence, quality, proximity, area, and number of green spaces within a 1.6 km of neighborhood center		[75]
**Greening intervention (*n* = 12)**			
	Proximity to cleaned-and-greened vacant lot	Proximity to untreated vacant lot	[13,14]
	Presence of emerald ash borer in Census block; number of trees removed	Absence of emerald ash borer in Census block	[16]
	Proximity to green stormwater infrastructure project	Proximity to green stormwater infrastructure wait-list control site	[15]
	Residence in one of 24 neighborhoods with green space improvements	Residence in one of 12 neighborhoods without green space improvements	[61]
	Residence within 2 blocks of cleaned-and-greened vacant lots	Residence within 2 blocks of untreated control vacant lots	[40]
	Residence in one of 20 neighborhoods with green space improvements; perceived greenery	Residence in one of 20 neighborhoods without green space improvements; perceived greenery	[20]
	Residence in greenway trail intervention neighborhood	Residence within 2 control neighborhoods without green space improvements	[42]
	Residence within 2-blocks of new planters	Residence within 2-blocks of new planters	[49]
	Walk (15 min) near vacant lots before after greening	Walks (15 min twice 3 months apart) near vacant lots	[50]
	Distance from residence to nearest city park (>1 hectare)	Distance from residence to nearest city park (>1 hectare)	[78]
	Residence in neighborhood with green space improvements and within 500 m of green space	Residence in neighborhood with no green space improvements	[60]
**Nature walk/exercise (*n* = 15)**			
	Walk (15 min) in urban green space	Walk (15 min) in urban built area	[69,70,71]
	Walks (30 min for 7 days) in urban park	Walks (30 min for 7 days) in urban street environment	[76,77]
	Walk (25 min) in urban green space	Walk (25 min) in urban shopping street; Walk (25 min) in commercial district	[54]
	Social urban park walks once/week for 6 weeks	Social club or swimming club once/week for 6 weeks	[56]
	Walk (90 min) in urban-area open space preserve	Walk (90 min) in built urban	[37]
	Walks (20 min twice per week for 8 weeks) in urban nature	Walks (20 min twice per week for 8 weeks) in built urban	[57]
	Run (4 miles) in urban park	Run (4 miles) in urban industrial area	[38]
	Walks (30 min on 3 consecutive days) in urban country park	Walks (30 min on 3 consecutive days) in urban built and blue settings	[58]
	Walk (40 min) in urban-area open space preserve	Walk (40 min) in urban area; relax in indoor setting	[43]
	Walk (30 min) in urban park	Walk (30 min) in urban setting	[59]
	Walk (9 min) in outdoor urban campus	Walk (9 min) indoors	[64]
	Viewing session (15 min), Walk (30 min) in urban park	Viewing session (15 min), Walk (30 min) in center city	[79]
**Nature leisure experience (*n* = 4)**			
	Outdoor sitting (20 min) in forested urban nature preserve	Outdoor sitting (20 min) in (1) tree-lined urban park, (2) urban plaza, or (3) outdoor shopping mall	[36]
	Visit to urban and peri-urban natural settings that vary as a function of biodiversity	Visit to urban squares with green elements, urban parks, pinewood forest plantations, and peri-urban natural protected areas	[80]
	Leisure time (85 min to 3 h) in urban park	Leisure time (2 hrs) in home	[44]
	30 min of outdoor gardening	30 min of indoor reading	[62]
	Daytime nature activities (2 weeks)	Daytime anagram puzzles (2 weeks)	[67]
**Residential relocation (*n* = 1)**			
	Perceived access to parks		[73]

**Table 3:** Health Outcome Measures with Study Paper Citations.

Health Outcome Measure	Citations	Health Outcome Measure	Citations
**Behavior**		**Metabolic**	
Behavioral problems	[33]	Abdominal obesity	[74]
Smoking	[78]	BMI	[22,24,48,51,57,78]
		Cortisol	[36,58,62,76,79]
**Birth Outcomes**		Diabetes	[74]
Birth weight	[39]	Prediabetes	[74]
Preterm birth	[39]		
Small for gestational age	[39]	**Mortality**	
		Acute ischemic stroke	[45,51]
**Cancer**		Any/all cause	[45,68,72]
Prostate cancer	[66]	Cancer	[45]
		Diabetes	[45]
**Cardiovascular**		Cardiovascular	[41,65,78]
Autonomic function	[57]	Cerebrovascular	[65]
Blood pressure - hypertension	[13,36,74,78]	Infections and parasitic disease	[45]
		Ischemic/coronary artery disease	[45,65]
Cholesterol	[13,36,78]	Kidney	[45]
CVD risk	[57]	Neurodegenerative disease	[45]
Diastolic blood pressure	[43,57,76,77]	Respiratory	[45,65,68]
Dyslipidaemia	[74]		
Heart rate (HR)	[43,50,57,69,70,71,76,77]	**Physical Activity**	
Heart rate recovery	[77]	Cycling frequency	[20]
Heart rate variability-ln(HF)	[57,58,69,70,71]	Exercise duration	[77]
Heart rate variability-ln(LF)	[58]	Exercise frequency	[13] [60]
Heart rate variability-ln(LF/HF)	[58,69,70,71]	MVPA	[21,23,25,26,27,28,29,31,32,34,52,59]
Heart rate variability-percent coefficient of component variance	[58]	Physical activity—duration	[78]
Peak diastolic blood pressure (DBP) during exercise	[77]	Taking up cycling for transport	[73]
Peak heart rate	[77]	Walking frequency	[20,42,61,75]
Peak systolic blood pressure (SBP) during exercise	[77]	Work load	[77]
Predicted aerobic fitness	[57]	Physical Health	
Pulse wave velocity	[77]	Physical health	[57]
Systolic blood pressure	[43,57,76,77]	Predicted aerobic fitness	[57]
**Mental Health**		**Mental Health**	
Anger	[38,43,69,70]	Sadness	[43]
Anxiety	[38,64,71]	Satisfaction	[60]
Anxious	[44]	Self-esteem	[56]
Attention	[17,46,47,58,79]	Stress	[13,36,64]
Attentiveness	[43]	Subgenual prefrontal cortex activation	[37]
Calm	[44]	Tired	[44]
Confusion	[38,69,70]	Total mood disturbance	[56]
Depression	[20,38,49,64,69,70]	Vigor	[38,69,70]
Elevating experience	[64]	Social capital	
Emotions	[38,54,71]	Sense of community	[49]
Energy	[44]		
Fatigue	[38,69,70]	**Violence-Aggression**	
Fear	[43]	Aggression	[35]
General health	[51,55,57,61,78]	Drug crimes	[13,16,36]
Happiness	[43]	Gun assault	[30]
Life functioning	[46,47]	Nuisance crimes	[13,16,36]
Mental health	[53,57]	Perceived safety	[40]
Motivation	[67]	Property crime	[14,16,36]
Nature connectedness	[67]	Violent crime	[13,14,16,36]
Negative affect	[47,62,64,67,76]		
Positive affect	[43,47,62,64,67,76]	**Respiratory**	
Quality of life	[78]	Aeroallergen Sensitization	[18]
Restoration	[36,58,79,80]	Allergic rhinitis	[18]
Rumination	[37,38]	Ear and nose symptoms	[18]
